# Treadmill Training Reduces Cerebral Ischemia-Reperfusion Injury by Inhibiting Ferroptosis through Activation of SLC7A11/GPX4

**DOI:** 10.1155/2022/8693664

**Published:** 2022-06-06

**Authors:** Tongye Liu, Yiteng Cui, Shanshan Dong, Xiangyi Kong, Xiangyu Xu, Yuyang Wang, Qi Wan, Qiang Wang

**Affiliations:** ^1^Department of Rehabilitation Medicine, The Affiliated Hospital of Qingdao University, China; ^2^Institute of Neuroregeneration & Neurorehabilitation, Department of Pathophysiology, Qingdao University, Qingdao 266071, China

## Abstract

The mechanism by which exercise training attenuates cerebral ischemia/reperfusion (I/R) injury, especially in the regulation of iron level in neuronal damage, has not been systematically studied. Here, we showed that treadmill training inhibited ferroptosis after I/R injury in rats. Modified neurologic severity score (mNSS) test showed that the motor function, reflex, and balance abilities in the I/R injury rats after treadmill intervention were significantly improved. Treadmill training decreased the level of lipid peroxides in the cerebral cortex of ischemic rats. We found that the protein levels of ferroptosis-related proteins including nuclear transcription factor E2-related factor 2 (Nrf2), cystine/glutamate reverse transporter (SLC7A11), and glutathione peroxidase 4 (GPx4) were decreased in rats after cerebral I/R injury, while treadmill training prevented the reduction of these proteins. Furthermore, we demonstrated that erastin- (a ferroptosis activator-) induced downregulation of SLC7A11 reversed the neuroprotective effect of treadmill training. This study provides the first evidence suggesting that treadmill training suppresses ferroptosis by activating the SLC7A11/GPx4 pathway, thereby protecting against cerebral I/R injury.

## 1. Introduction

Cerebral ischemia is a serious neurological condition and the main cause of adult disability in many countries [1]. Ischemic stroke accounts for the vast majority of stroke cases [2]. Oxidative stress occurs after cerebral ischemia, which involves an increased level of ROS [3, 4]. In diseases of the nervous system, abnormally high iron content leads to oxidative stress injury. In addition, high Fe^[2+]^ (ferrous) content in cells promotes the production of ROS, which affects the balance between ROS production and clearance and causes the excessive increase of hydroxyl free radicals, eventually resulting in cell death [5]. Therefore, reducing the abnormal iron content in the brain is one of key challenges in the treatment of cerebral ischemia.

Ferroptosis was first described in 2012 as a new programmed cell death pathway different from apoptosis [6]. Its remarkable feature is the increase in iron-dependent ROS lipid peroxidation. After ferroptosis, the activity of GPx4 (glutathione peroxidase 4) and GSH (glutathione) synthesis is inhibited, which can reduce the level of phospholipid peroxide. When GPx4 activity decreases, it causes the accumulation of lethal ROS, thereby resulting in cell death [7, 8]. In addition, xCT/SLC7A11 (cystine/glutamate reverse transporter) is a key regulatory molecule in the process of ferroptosis [9]. SLC7A11 exchanges extracellular cystine with intracellular glutamate as the source of GSH, the main antioxidant in cells. SLC7A11 can inhibit lipid peroxidation and ferroptosis [10]. Recently, it has been found that many diseases are related to ferroptosis [11]. For example, ferroptosis is associated with harmful ROS and iron content in stroke [12]. Liu and colleagues showed that an apoptosis-stimulating protein inhibitor of p53 can reduce acute lung injury caused by intestinal ischemia/reperfusion by inhibiting ferroptosis [13]. In addition, oxidative stress induces ferroptosis disease and mitochondrial dysfunction. The study found that ACSl4 aggravated stroke by promoting ferroptosis-induced brain injury [14]. Ferroptosis has been detected after cerebral ischemia, but the mechanism of ferroptosis regulation in cerebral ischemia is still unclear.

Aerobic exercise is a way to promote cardiovascular health [15]. It is recommended in healthy people as well as in specific situations such as stroke. It is defined as any activity that uses large muscle groups and is rhythmic and sustainable. Aerobic exercise is beneficial for all stages of stroke, including acute, subacute, and chronic, and should be included in the whole process of stroke rehabilitation [16, 17]. However, many neuroprotective mechanisms of physical training are still poorly understood. In this study, we examined whether treadmill training can improve the recovery of neurological function after ischemia/reperfusion injury in rats by reducing ferroptosis and the underlying molecular mechanism.

## 2. Materials and Methods

### 2.1. Animals

Two-month-old adult male Sprague Dawley rats were purchased from Qingdao Peng Yue Animal Husbandry Co. and were kept under a 12-hour light/dark cycle in the same colony room, with appropriate temperature. The animals were randomly assigned into the following three groups: (1) sham group (*n* = 7): rats that received surgery without middle cerebral artery occlusion (MCAO); (2) MCAO group (*n* = 7): rats that received middle cerebral artery occlusion; and (3) MCAO + Treadmill group (*n* = 7): rats that underwent early treadmill training after MCAO. To study the effect of treadmill training on ferroptosis induced by I/R (ischemia/reperfusion) injury, we treated MCAO rats with erastin (10 mg/kg/day; MedChemExpress) every day for 20 days; then, the rats were divided into those that would undergo treadmill training and those without daily training for another 14 days. We dissolved erastin in 5% DMSO + corn oil by gently shaking in a water bath at 37°C (MedChemExpress). The animals were randomly divided into four groups: (1) sham group (*n* = 7): rats that underwent surgery without MCAO; (2) MCAO group (*n* = 7): rats that received middle cerebral artery occlusion; (3) MCAO + Treadmill group (*n* = 7): rats that underwent extra treadmill training; (4) MCAO + Treadmill + Erastin group (*n* = 7): rats that underwent extra treadmill training and received erastin. The study was reviewed and approved by the Ethics Committee of the Affiliated Hospital of Qingdao.

### 2.2. MCAO Model

The model of cerebral ischemia in rats was established by the suture occlusion technique [18]. Male Sprague Dawley rats weighing 250–300 g were anesthetized with a mask with 4% isoflurane in 70% NO_2_ and 30% O_2_. During and after the surgical procedure, the temperature of the rats was maintained at 37°C using an electric blanket. A midline incision was made in the neck area to dissect the right external carotid artery (ECA). We carefully inserted a 3-0 monofilament nylon wire from ECA into the right internal carotid artery to block the starting point of the right middle cerebral artery (MCA). After arterial occlusion for 1.5 hours, the monofilament nylon suture was pulled out to restore the blood flow, and then, the wound was sutured. Sham-operated mice underwent the same surgery, except for inserting and removing the nylon wires.

### 2.3. Treadmill Training

The exercise program used throughout the study was slightly adjusted compared with the methods used in previous studies [19]. In brief, three days before the operation, the rats adapted to the treadmill training environment and continued at a speed of 1–2 m/min for 20 minutes. To ensure that the rats would be able to perform normal training in the next training, we eliminated the rats that did not actively participate in the training. On the second day after the operation, the training group did not have any complications and began treadmill training. The cerebral ischemia group did not receive training and received routine feeding in the above environment for two weeks. Compared with the MCAO group, the MCAO + Treadmill group received training for 14 days. The training time was set at 30 min/day; the training speed on the first day was set at 6 m/min and then at 10 m/min every day. Similar to the above training methods, the MCAO + Treadmill training + Erastin group began exercise training after 20 days of erastin injection for 14 days.

### 2.4. Behavioral Test

mNSS (modified neurologic severity scores) is a comprehensive test of movement, sensation, balance beam, reflex, and abnormal movement [20]. Neurological function was scored from 0 to 14 (normal score, 0; maximum defect score, 14). Higher scores indicated more serious damage.

The hanging wire test mainly evaluates the grasping power of rats [21]. The experimental rats were placed on the cage cover; when they grasped the cage cover tightly, we turned the cage over quickly and recorded the time when the hind limbs released the cage. In all of the animals, behavioral tests were performed 1, 7, and 14 days after MCAO or erastin treatment.

### 2.5. Oxidative Stress Measurement

To examine whether aerobic exercise therapy reduces lipid peroxidation injury, the levels of enzyme activity of glutathione peroxidase (GSH-Px), GSH, and malondialdehyde (MDA) were evaluated using different test kits in accordance with the manufacturer's instructions (Nanjing Jian Cheng Bioengineering Institute). For biochemical analysis, regions corresponding to the penumbra from the right (ipsilateral) were dissected according to previous studies [22, 23]. The penumbra (adjacent cortex) was separated from the core by transverse diagonal cuts at approximately the “2 o'clock” and “5 o'clock” positions. After weighing a part of the penumbra area to prepare tissue homogenate (10% *w*/*v*), the penumbra tissue was diluted in cold normal saline. The absorbance value of reduced glutathione was read at 405 nm. GSH content was expressed as nmol/mg protein. To calculate the MDA level of core tissue, the absorbance value of each tube was measured at 532 nm, and the concentration of MDA was expressed as nmol/mg protein. The activity of GSH-Px was detected by quantitative oxidation of reduced glutathione to oxidized glutathione. The activity of GSH-Px was calculated by measuring the OD level at 412 nm using a microplate reader.

### 2.6. Iron Measurement

The penumbra tissue was immediately diluted in cold saline to prepare tissue homogenate (10% *w*/*v*). The supernatant was collected after centrifugation. In line with the manufacturer's instructions, we put the mixed liquid into boiling water bath for five minutes and cooled it with running water. After centrifugation, we took the supernatant and measured the absorbance value of each tube. The iron content was measured using the iron analysis kit (Nanjing Jian Cheng Bioengineering Institute).

### 2.7. Immunoblot Analysis

Cortical penumbra tissue was removed from the brain and stored at −80°C until use for western blotting. Western blot analysis was performed as previously described [18]. Briefly, the membrane was blocked with 5% milk in Tris-buffered saline at room temperature for 60 minutes. The membranes were incubated with primary and secondary antibodies. The membranes were observed by a western blot analysis and chemiluminescence detection system (simple protein). The ImageJ (Rawak Software, Germany) software was used to quantify the western blot data. Primary antibodies included GPx4 (1: 1000, Abcam, Cambridge, MA, USA), NRF2 (1 : 1000, Abcam, Cambridge, UK), SLC7A11 (1: 1000, Abcam, Cambridge, UK), and *β*-actin (1 : 5000 Abcam, Cambridge, UK). Secondary antibodies, including goat anti-rabbit IgG-HRP and goat anti-mouse IgG-HRP, were from Abcam.

### 2.8. Immunofluorescence

Rat brain tissue was placed in a frozen microtome. The thickness of coronal slice was set at 20 *μ*m, and the brain slices were placed on slides. The brain slices were fixed with 4% paraformaldehyde for 10 minutes, and then, the membranes were broken with 0.3% Triton X100. Brain slices were blocked with 10% goat serum for 60 minutes and then incubated with the primary antibody (SLC7A11 and GPx4) at 4°C overnight. After washing in PBS, the brain slices were incubated with Alexa Fluor 555 and 488 secondary antibodies (Abcam) at room temperature for one hour. We used a sealing agent for sealing. Photographs were then taken with a confocal microscope.

### 2.9. Statistical Analysis

Graphics were drawn and statistically analyzed by the GraphPad Prism 7.0 software. All data were expressed as mean ± SEM. One-way analysis of variance (ANOVA) or two-way ANOVA was used for statistical analysis of different groups, followed by Tukey's multiple-comparison post hoc test. *p* < 0.05 was considered statistically significant.

## 3. Results

### 3.1. Treadmill Training Improves the Neurological Deficit in Rats after the Cerebral I/R Injury

**Figure 1:**
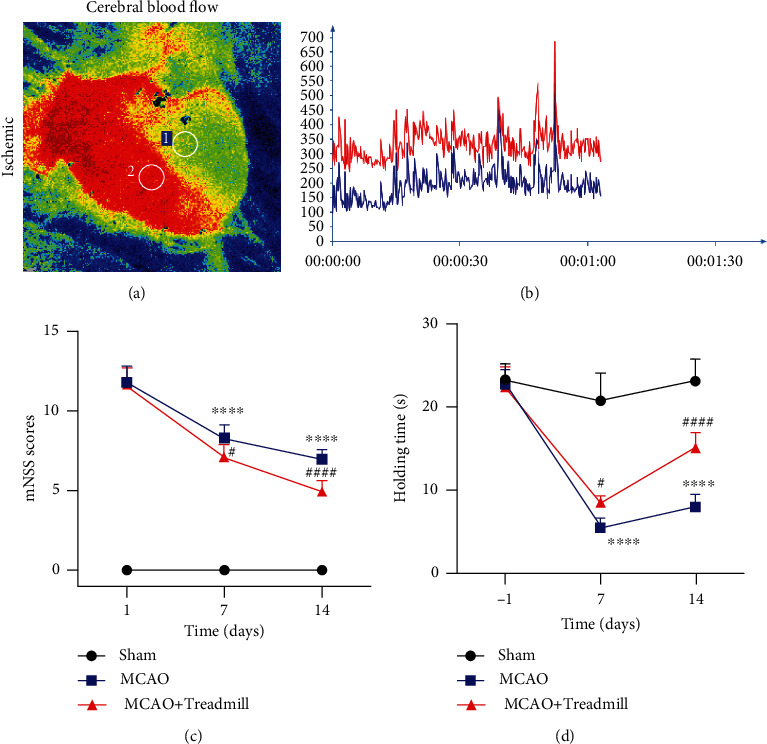
Treadmill training attenuated the neurological deficit in cerebral I/R (ischemia/reperfusion) injury-operated rats. (a) Representative 2-D laser speckle images of MCAO rat. (b) Real-time monitoring of blood flow in left and right cerebral cortex of MCAO rats. (c) Compared with the sham operation group, the neurological deficit score in the MCAO group was significantly higher. After exercise training, the neurological deficit score in the treadmill training group was significantly lower (*n* = 7 for sham, MCAO, and MCAO + Treadmill rats). (d) Compared with the sham operation group, the grasping power in the MCAO group was significantly lower. After exercise training, the grasping power was significantly higher (*n* = 7 for sham, MCAO, and MCAO + Treadmill rats). ^∗∗∗∗^*p* < 0.0001 vs. sham; ^#^*p* < 0.05 vs. MCAO; ^####^*p* < 0.0001 vs. MCAO.

We performed cerebral cortical blood flow imaging to ensure the success of modeling in each rat. Laser speckle blood flow detection showed that the cerebral blood flow on the infarct side decreased after modeling (Figures 1(a) and 1(b)). mNSS was used to evaluate the motor function, balance, and reflex function in the rats after the I/R, and the hanging wire test was used to evaluate the grasping power of the rats. Compared with the sham operation group, the neurological deficit score in the MCAO group was significantly higher, and the grasping power in the MCAO group was significantly lower. After exercise training, the neurological deficit score in the treadmill training group was significantly lower, and the grasping power was significantly higher (Figures 1(c) and 1(d)). These results show that treadmill training can reduce the damage caused by I/R.

### 3.2. Treadmill Training Decreases the Levels of Lipid Peroxidation and Iron Ions in the Rat Cortical Penumbra after the I/R Injury

**Figure 2:**
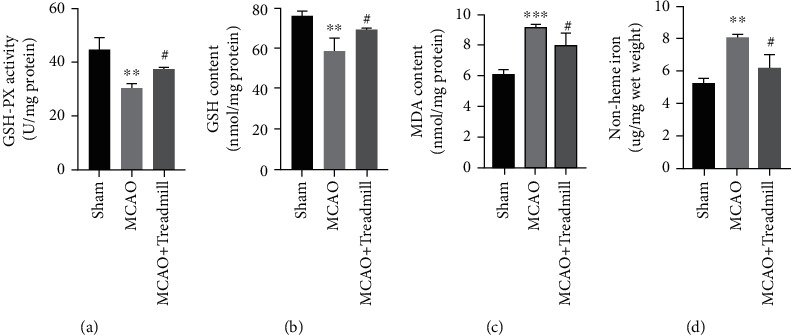
Effect of treadmill training on stroke-induced lipid peroxidation and iron ion expression in the cortical penumbra. (a–d) The effects of treadmill training on activities of GSH-PX and levels of GSH, MDA, and iron from different groups, respectively (*n* = 7 for sham, MCAO, and MCAO + Treadmill rats). ^∗∗^*p* < 0.01 vs. sham; ^∗∗∗^*p* < 0.001 vs. sham; ^#^*p* < 0.05 vs. MCAO.

To examine whether treadmill training had a protective effect on neuron injury through an antioxidant mechanism, we evaluated biomarkers of oxidative damage. Our results showed that antioxidant enzyme activities, including GSH-Px and GSH levels, of the model rats decreased and MDA levels increased compared with the sham operation group. Treadmill training significantly reversed these changes (Figures 2(a)–2(c)). Similar to the above results, the level of iron ion is another important factor affecting health. There was a significant increase in iron in the MCAO group but not in the sham operation group or in the treadmill treatment group (Figure 2(d)). These findings suggest that I/R injury causes ferroptosis, and the protective effect of treadmill training may be related to the inhibition of ferroptosis.

### 3.3. Treadmill Training Suppresses Ferroptosis in the Cortical Penumbra of Rats after the Cerebral I/R Injury

**Figure 3:**
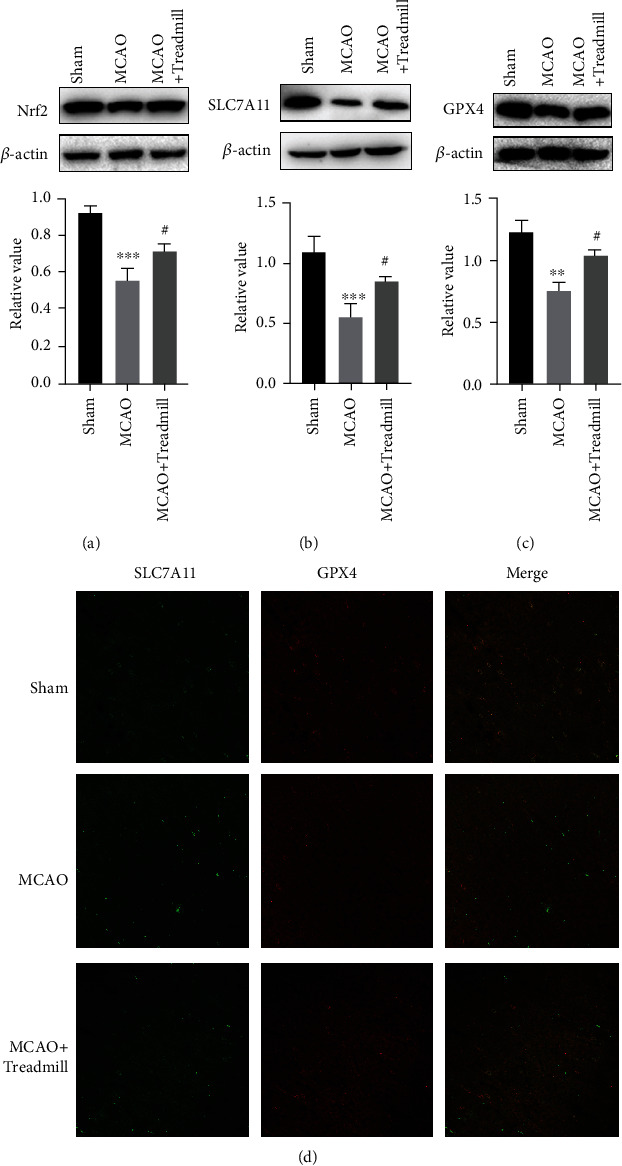
Treadmill training altered the protein expression of Nrf2, SLC7A11, and GPx4 of the cortical penumbra in cerebral I/R injury rats. (a–c) The quantitative analysis of the protein levels of Nrf2, SLC7A11, and GPx4 in rat cortical penumbra. The data are expressed as the mean ± SEM (*n* = 7 for sham, MCAO, and MCAO + Treadmill rats). (d) Brain sections were stained for SLC7A11 (green) and GPX4 (red) in the ischemic cortex subjected to reperfusion for 14 d or under sham control conditions or treadmill control conditions. ^∗∗^*p* < 0.01 vs. sham; ^∗∗∗^*p* < 0.001 vs. sham; ^#^*p* < 0.05 vs. MCAO.

To examine the effect of treadmill training on the penumbra iron ion level, the protein expression levels of Nrf2, SLC7A11, and GPx4 were detected. The results showed that the expression levels of Nrf2, GPx4, and SLC7A11 decreased in the MCAO group compared with the sham operation group. However, treadmill training increased the protein expression levels of NRF2, SLC7A11, and GPx4 in the model rats (Figures 3(a)–3(c)).

The expression of SLC7A11 and GPx4 was further evaluated by immunofluorescence. Consistent with the protein levels of SLC7A11 and GPx4, I/R injury significantly reduced the expression levels of SLC7A11 and GPx4, which was reversed by treadmill training (Figure 3(d)). Our results suggested that treadmill training can inhibit ferroptosis in the penumbra of MCAO rats.

### 3.4. Erastin-Induced Ferroptosis Promotes the Neurological Deficit of Rats after Cerebral I/R Injury

**Figure 4:**
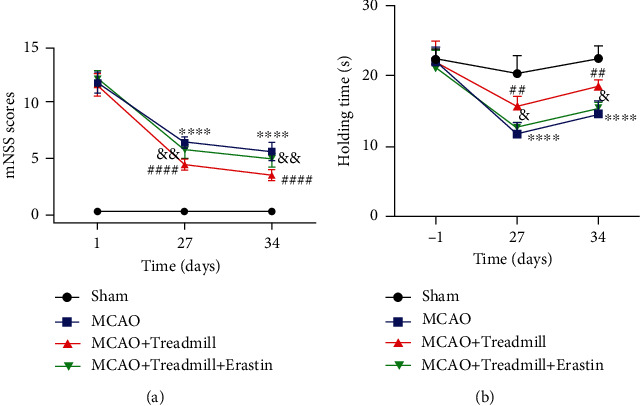
Erastin-induced ferroptosis promotes the neurological deficit in cerebral I/R injury rats. (a) Compared with the MCAO + Treadmill group, the neurological deficit score in the MCAO + Treadmill + Erastin group was significantly higher. (b) Compared with the MCAO + Treadmill group, the grasping power in the MCAO + Treadmill + Erastin group was significantly lower (*n* = 7 for sham, MCAO, MCAO + Treadmill, and MCAO + Treadmill + Erastin rats). ^∗∗∗∗^*p* < 0.0001 vs. sham; ^##^*p* < 0.05 vs. MCAO; ^####^*p* < 0.0001 vs. MCAO; ^&^*p* < 0.05 vs. MCAO + Treadmill; ^&&^*p* < 0.01 vs. MCAO + Treadmill.

Erastin activates lipid peroxidation, induces ferroptosis, a nonapoptotic form of cell death, and seems to promote iron ions. It is an inhibitor of SLC7A11. Our results showed that compared with the MCAO group, the neurological deficit score in the treadmill training group was significantly lower, and the grasping power was significantly higher. However, the effect was reversed in the MCAO + Treadmill + Erastin group (Figures 4(a) and 4(b)). These results suggest that erastin can reverse the neuroprotective effect of treadmill training in rats after I/R injury.

### 3.5. Treadmill Training Suppresses Ferroptosis by Activating the SLC7A11/GPx4 Axis

**Figure 5:**
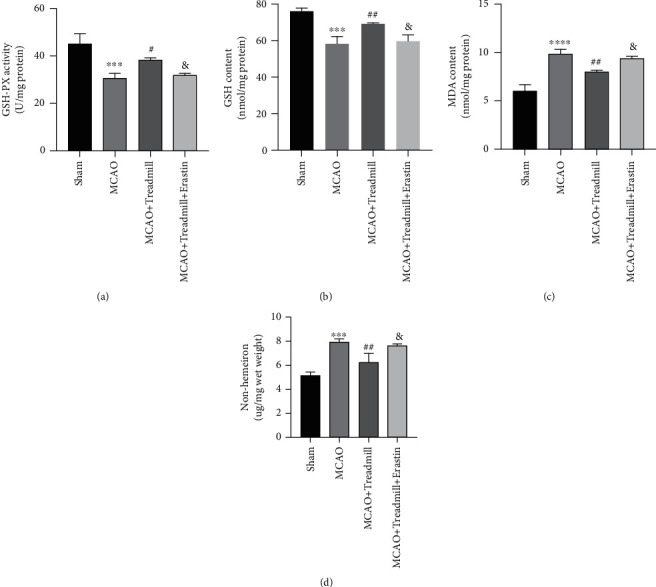
Erastin-induced ferroptosis reverses the antilipid peroxidation effect of treadmill training. (a–d) The effects of erastin on activities of GSH-PX and levels of GSH, MDA, and iron from different groups, respectively. Treadmill training could inhibit the lipid peroxidation induced by cerebral I/R. However, when SLC7A11 was inhibited, treadmill training inhibiting ferroptosis effects could be partially repealed. ^∗∗∗^*p* < 0.001 vs. sham; ^∗∗∗∗^*p* < 0.0001 vs. sham; ^#^*p* < 0.05 vs. MCAO; ^##^*p* < 0.05 vs. MCAO; ^&^*p* < 0.05 vs. MCAO + Treadmill.

**Figure 6:**
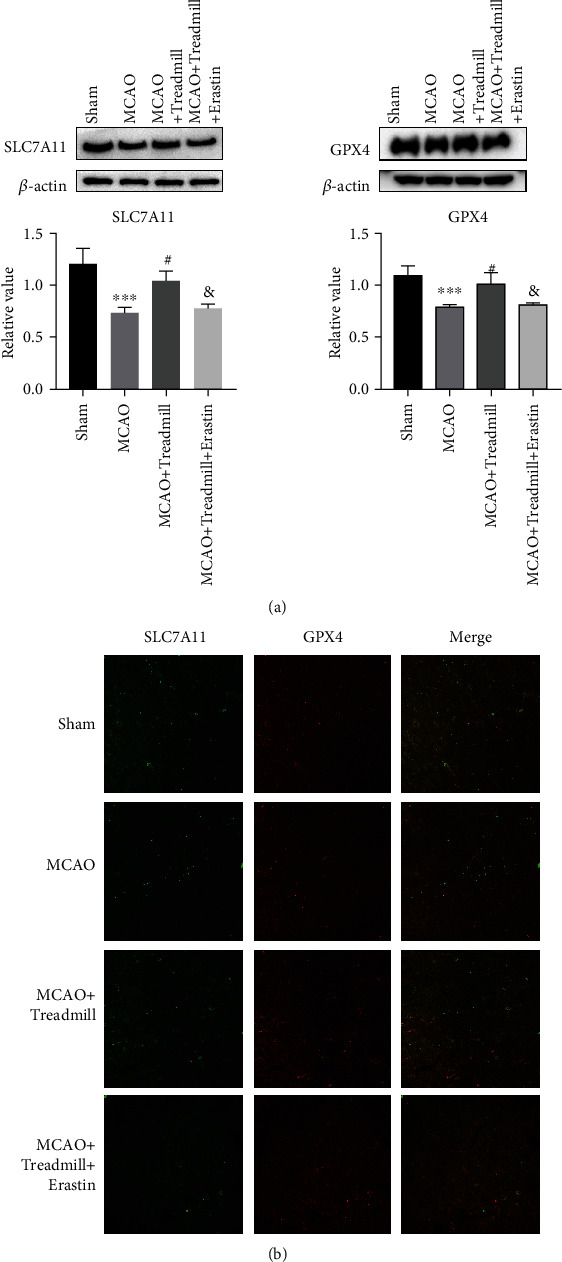
(a) Representative immunoblot and quantification of SLC7A11 and GPX4 in the cortical penumbra after I/R injected with erastin for 20 days (10 mg/kg/day). *β*-Actin was used as a loading control. (b) Confocal microscopy of the immunofluorescence staining of brain sections with SLC7A11 (green) and GPX4 (red) after subjected to reperfusion for 14 d or under sham conditions or treadmill control conditions or treadmill + erastin control conditions. The data are means ± SD. ^∗∗∗^*p* < 0.001 vs. sham; ^∗∗^*p* < 0.01 vs. sham; ^#^*p* < 0.05 vs. MCAO; ^&^*p* < 0.05 vs. MCAO + Treadmill.

To investigate the role of the SLC7A11/GPx4 pathway activation in the effect of treadmill exercise on cerebral ischemic injury, we examined the expression of SLC7A11 and GPx4 by western blot. The expression of SLC7A11 GPx4 protein was inhibited in the MCAO model, but the expression of GPx4 protein was significantly increased in MCAO rats with treadmill training. We further inhibited SLC7A11 with erastin to confirm that the neuroprotective effect of treadmill training occurred through SLC7A11. Our results showed that compared with the MCAO group, treadmill running significantly reduced MDA and iron ions, increased the expression of GSH and GSH-Px, and increased the protein expression levels of SLC7A11 and GPX4. However, the effect was reversed in the MCAO + Treadmill training + Erastin group (Figures 5(a)–5(d) and 6(a)). Immunofluorescence further evaluated the expression of SLC7A11 and GPx4; the results were consistent with those of western blot, but these effects were reversed by inhibiting SLC7A11 (Figure 6(b)). Taken together, these findings suggest that treadmill protects neurons by activating the SLC7A11/GPx4 pathway.

## 4. Discussion

There is increasing evidence that early exercise training is beneficial for the treatment of cerebral ischemia [24]. Moreover, it has been reported that treadmill running can regulate cerebral edema, apoptosis, oxidative damage, stem cells, and other mechanisms, so as to play a neuroprotective role in the brain [25]. However, it is not clear whether exercise training can act on ferroptosis, and if so, what the relevant mechanism is. In this study, we demonstrated that treadmill therapy significantly inhibited neurological deficits in rats exposed to I/R injury. In addition, our data also showed that treadmill training was able to inhibit ferroptosis in rats with cerebral I/R injury.

The measurement of behavioral changes showed that cerebral I/R led to defects in motor function, balance, and reflex function and finally to the impairment of neural function. Through the mNSS score and the hanging wire test score, we showed that treadmill training partially reversed the motor ability, balance ability, and reflex function of the model group and also reduced the neurological dysfunction. The previous studies have shown that treadmill training can reduce the mNSS score and enhance the grasping ability based on the hanging wire test. Physical training can improve the motor function and promote the recovery of neurological function after stroke [26, 27]. The behavioral performance in this study is consistent with the opinion above.

Abnormal deposition of iron ions in ischemic stroke may cause ferroptosis—iron-dependent cell death [28]. So far, the mechanism of ferroptosis is not completely clear, but it is known that the abnormal deposition of iron, lipid peroxidation, and fatty acid accumulation is the signs of ferroptosis [29, 30]. Importantly, previous studies have shown that pharmacological selenium supplements can inhibit ferroptosis and treat stroke [12, 31]. The mechanism of ferroptosis is involved in the mechanism of ischemic stroke [32]. It has been reported that many ferroptosis inhibitors can play a neuroprotective role in neurodegenerative diseases [33, 34]. Some studies have found that exercise training can change the expression level of ferroptosis genes in rats with intracerebral hemorrhage [19]. Therefore, exercise training may be a nonpharmacological treatment of cerebral ischemia, and we should consider whether exercise training can inhibit ferroptosis in cerebral ischemia. The results of lipid peroxidation biomarkers (GSH-Px, GSH, MDA, nonheme iron) showed that lipid peroxidation increased after cerebral I/R injury and decreased after treadmill training, indicating that exercise training affected ferroptosis after cerebral ischemia. As described above, the expression levels of intracellular antioxidant enzyme GPx4, SLC7A11/XCT, and Nrf2 increased in the brain tissue of rats after exercise training. GPx4 can reduce cytotoxic lipid peroxide to the corresponding alcohol; thus, inhibiting GPx4 activity could lead to the accumulation of lipid peroxides in the cell membrane [35, 36]. System XC is an important antioxidant system in cells. System XC exchanges intracellular glutamate for extracellular cystine (cys2) at a 1 : 1 ratio. Cystine can synthesize glutathione, and XCT reduced activity affects the synthesis of GPx4 [37–39]. Nrf2 can regulate many ferroptosis transcriptional genes, including GSH [40]. Therefore, after exercise training, the expression levels of GPx4, Nrf2, and SLC7A11 increased, which likely increased the antioxidant capacity of cells in the brain cortex and reduced ferroptosis in the rat brain.

Ferroptosis was first described by Stockwell in 2012 as a form of cell death different from apoptosis [6]. It is characterized by increased lipid peroxidation caused by iron-dependent ROS [41]. The main mechanism involves the inhibition of cystine/glutamate reverse transporter to promote iron death, iron transporter, and iron “overload” and inhibit GPx4-induced ferroptosis, regulatory pathways of other enzymes, and fatty acid accumulation [42–44]. Unexpectedly, when we studied the neuroprotective effect of treadmill training after I/R-induced neuronal injury, we found that the key inhibitory mechanism of ferroptosis included the SLC7A11/GPx4 pathway. In addition, we found that adding a ferroptosis promoter increased the level of lipid peroxidation and decreased the level of ferroptosis-related proteins, which hindered the protective effect of exercise on stroke. It was further confirmed that the SLC7A11/GPx4 pathway inhibited ferroptosis and played a protective role after cerebral ischemic injury. Our data analysis was limited to the effect of the SLC7A11/GPx4 axis on ferroptosis, and the contribution of other mechanisms was not investigated. More research is needed on the pathogenesis of ferroptosis, and a deeper understanding of ferroptosis will certainly be beneficial for the treatment of related diseases.

In conclusion, our results showed that the protective effect of treadmill training on rats exposed to I/R injury is related to the antiferroptosis effect and that it might be mediated at least in part by the SLC7A11/GPx4 axis.

## Data Availability

The data used to support the findings of this study are available from the corresponding authors upon request.
